# Evaluation of Confidence and Knowledge Regarding Chest Compression Training Among Children at a Health Promotion Event

**DOI:** 10.7759/cureus.68412

**Published:** 2024-09-01

**Authors:** Masahiko Sakamoto, Takahisa Ogawa, Junpei Hasumi

**Affiliations:** 1 Pediatrics, Saku Central Hospital Advanced Care Center, Saku, JPN; 2 Orthopedics, Saku Central Hospital Advanced Care Center, Saku, JPN

**Keywords:** hospital festival, public education, health promotion, children, early cpr training

## Abstract

Introduction

Bystander cardiopulmonary resuscitation (CPR) significantly increases out-of-hospital cardiac arrest survival rates. Early CPR implementation and public education are essential for everyone, including children. Despite challenges such as ensuring proper chest compression depth in children, early CPR training at school is beneficial and supported by various studies.

However, few studies demonstrate the effectiveness of CPR training for children in environments outside of school, such as health festivals. We conducted chest compression training for children under 15 years who attended a hospital festival and evaluated the changes in their confidence and knowledge of the training.

Methods

This study evaluated chest compression training in children under 15 years old at a hospital festival. Participants included preschoolers to junior high school students. Training effectiveness was assessed using pre- and post-training questionnaires to measure confidence in and knowledge of chest compressions. Data were analyzed using the χ² test to compare responses before and after training, with a p-value < 0.05 considered statistically significant. The training involved a 10-minute hands-only session using CPR mannequins conducted by paramedics, doctors, and nursing students.

Results

The study included 180 children (51% male, 49% female). Only 14% had prior CPR training. After CPR training, significant improvements in confidence and knowledge regarding chest compressions were observed among preschool and elementary school students. The overall correct response rate increased from 51% to 97% for the location and from 59% to 92% for the depth of chest compressions.

Discussion

This study evaluated CPR training for children at a hospital festival and showed that few children have experience with CPR training. Our CPR training improved children's confidence and knowledge of chest compressions. Despite concerns about the strength of younger children in effective chest compressions, preschool, and elementary school children showed high motivation and capability. This study highlights the importance of early CPR education supported by both school and health festival initiatives. The limitations include the short training duration and lack of automated external defibrillator instructions.

Conclusion

We confirmed that CPR training at a hospital festival improves children's knowledge and confidence. Future large-scale studies are needed to evaluate long-term retention and broader age ranges.

## Introduction

Out-of-hospital cardiac arrest (OHCA) is the third leading cause of death in developed countries [1]. Even if survival is achieved, reduced blood circulation can cause brain damage [2]. While the survival rate after OHCA varies significantly by region, it is less than 1% in China and only 8% in Western countries, indicating a very low overall survival rate [3]. Cardiopulmonary resuscitation (CPR) plays a crucial role in maintaining blood circulation and delaying brain damage [4]. To improve the survival rates of patients with OHCA, the importance of early CPR implementation and public CPR education are emphasized [2]. The implementation of bystander CPR can increase survival rates from cardiac arrest by up to two to four times [5].

Therefore, enhancing CPR education in children is crucial. Several studies have demonstrated the importance of CPR training during childhood. For example, learning CPR at an early age has been suggested to improve response capabilities during emergencies [6]. Furthermore, children who acquire knowledge of lifesaving measures are more likely to participate in rescue activities even after becoming adults [7]. Children are also expected to disseminate lifesaving measures by spreading knowledge to their families and communities [6].

The effectiveness of CPR training in children has been verified, showing that they can acquire basic CPR skills through adequate training. For example, CPR education for elementary school students in South Korea significantly improves their knowledge, attitude, self-efficacy, and confidence in CPR after training [8]. Systematic reviews have also demonstrated the effectiveness of CPR training in school environments, indicating that continuous training enhances students’ CPR knowledge and skills [9].

However, challenges remain, such as ensuring appropriate chest compression depth in children [7]. In general, effective chest compressions require a body weight of about 45 kg, which corresponds to individuals aged 13 and older. A survey conducted among teachers in Japan revealed that most believed that the appropriate age to start CPR training is from upper elementary school to junior high school [10], indicating that early CPR training for children has not been widely implemented.

We believe that early CPR training is important. Teaching the importance of life through training is possible in lower elementary schools. Hori et al. reported that CPR training for elementary school students resulted in higher satisfaction compared to older students [11]. Repeated CPR training for preschool children is also considered effective, and the European Resuscitation Council (ERC) recently recommended that all school-aged children learn CPR [12]. However, there are few reports on the effects of training on younger children.

Since 1947, our institution has held annual hospital festivals for local residents. The hospital festival aims to disseminate medical and health knowledge. Many events have also been conducted to attract children from various age groups. Health education festivals play an important role in spreading health information and raising awareness among residents and are frequently used in community education on cancer screening [13, 14].

A few studies on CPR training have been conducted at health festivals. For example, a study conducted on CPR training modules during a three-day music festival reported that six months after the training, participants showed increased willingness, knowledge retention, and awareness of performing CPR on strangers [15]. Another approach introduced face-to-face instructions in small groups to teach adult basic life support to festival attendees [16]. However, to date, no study has applied this approach to young children and elementary school-aged children.

In this study, we set up a hands-only CPR booth for children under the age of 15 years who attended our hospital festival and conducted chest compression training. We aimed to evaluate changes in the participants' confidence in and knowledge of chest compressions before and after the training.

## Materials and methods

Ethical considerations

This study was approved by the Ethics Review Committee of Saku Central Hospital (Approval No.: R202405-04). We obtained verbal consent for study participation from the child's parent/guardian when the child participated in the hands-only CPR booth.

Study participants and data collection methods

**Figure 1:**
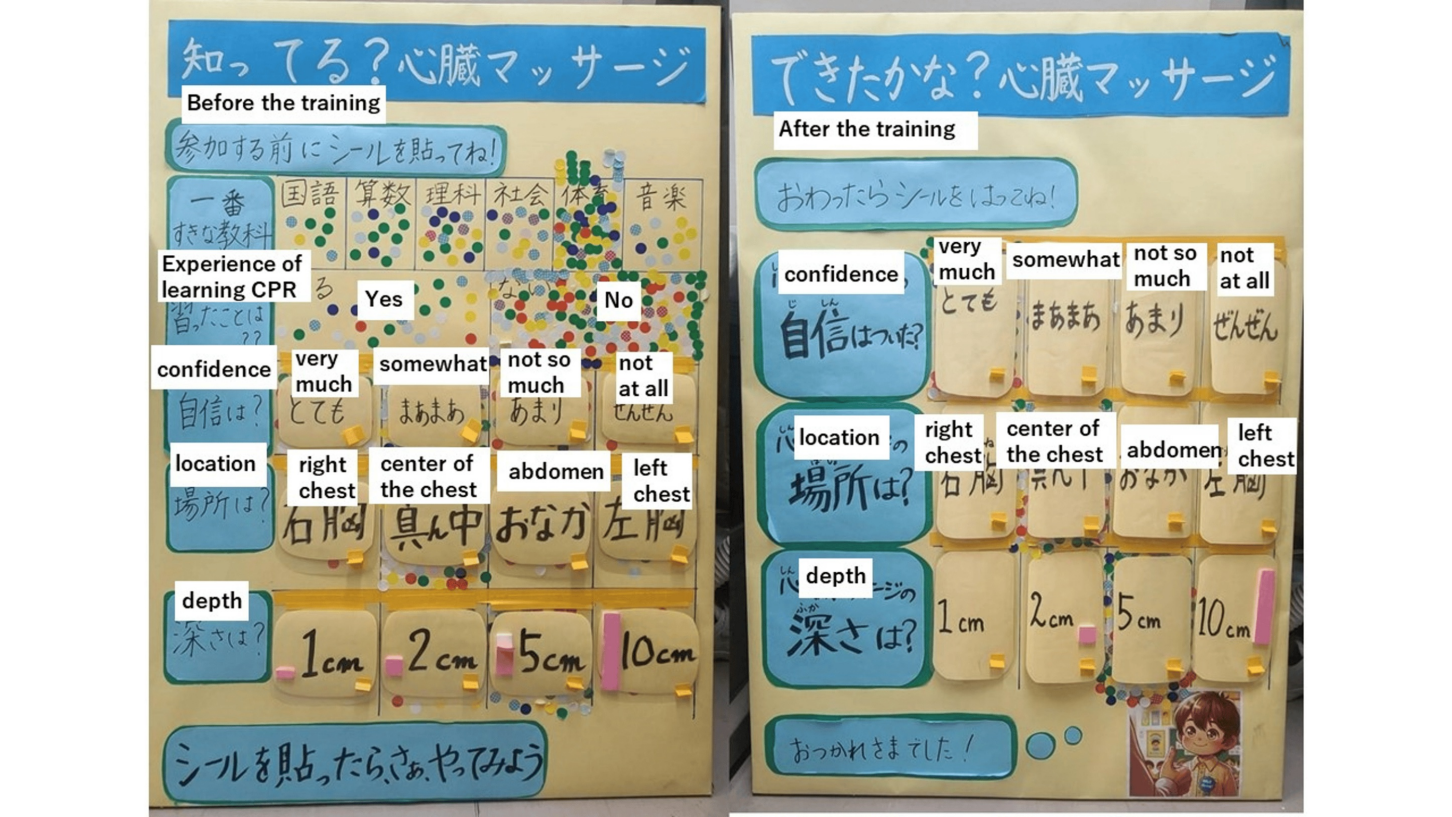
The appearance of the actual response boards (left: before the training, right: after the training)

This study targeted children of junior high school age and younger who visited the hands-only CPR booth at a hospital festival held at Saku Central Hospital on May 19, 2024. Children and their parents received a briefing on CPR training from the attending nurse and were given a sticker if both children and their parents agreed to participate. The stickers were color-coded according to sex (male, female) and school-age group (preschoolers, lower elementary school students, upper elementary school students, and junior high school students), resulting in six categories. Before the training, the participants answered questions regarding chest compressions. To avoid the influence of other participants' responses, an opaque cover was used in the area where the stickers were to be placed, ensuring that participants could not see the others' answers beforehand (Figure 1). After receiving the instructions, the participants answered the same questions they had been asked before the instructions, completing their participation in the study. Participants who were older than senior high school age were excluded from the study.

Training description

The training was conducted in a dedicated training space within the hospital building where the festival was held. The training space displayed panels summarizing the key points of chest compressions based on the Japan Resuscitation Council (JRC) Resuscitation Guidelines.

The training was carried out in two booths using standard adult resuscitation manikins commonly used in medical simulation training. Each booth was staffed by nursing students and paramedics as instructors, who provided individualized, hands-only CPR training under the supervision of a pediatrician. The training lasted for 10 minutes and was conducted in accordance with the JRC Resuscitation Guidelines.

The learning objectives of this training were to master the correct depth, location, and frequency of chest compressions.

Survey questionnaire

The participants were asked about their experience of chest compression training before the training session. Before and after training, they responded to questions regarding their confidence in and knowledge of chest compressions with stickers on the corresponding panel. Confidence was evaluated using a four-point Likert scale (very confident, somewhat confident, not very confident, not confident at all). Knowledge questions pertained to the location and depth of chest compression, with four options for each (left chest, right chest, center of the chest, abdomen) and (1 cm, 2 cm, 5 cm, 10 cm), respectively, and were categorized as correct or incorrect. Specifically, the correct location for chest compression was categorized as "center of the chest" and others as incorrect, and the correct depth as "5 cm" and others as incorrect.

Data analysis and statistics

Categorical variables were presented as frequencies and percentages. The χ² test was used to compare responses to questions before and after the training, evaluating changes in confidence and knowledge regarding CPR. Statistical significance was set at p < 0.05. significant. Data analysis was performed using the Stata 17 software (StataCorp LP, College Station, Texas).

## Results

Participant characteristics

**Figure 2:**
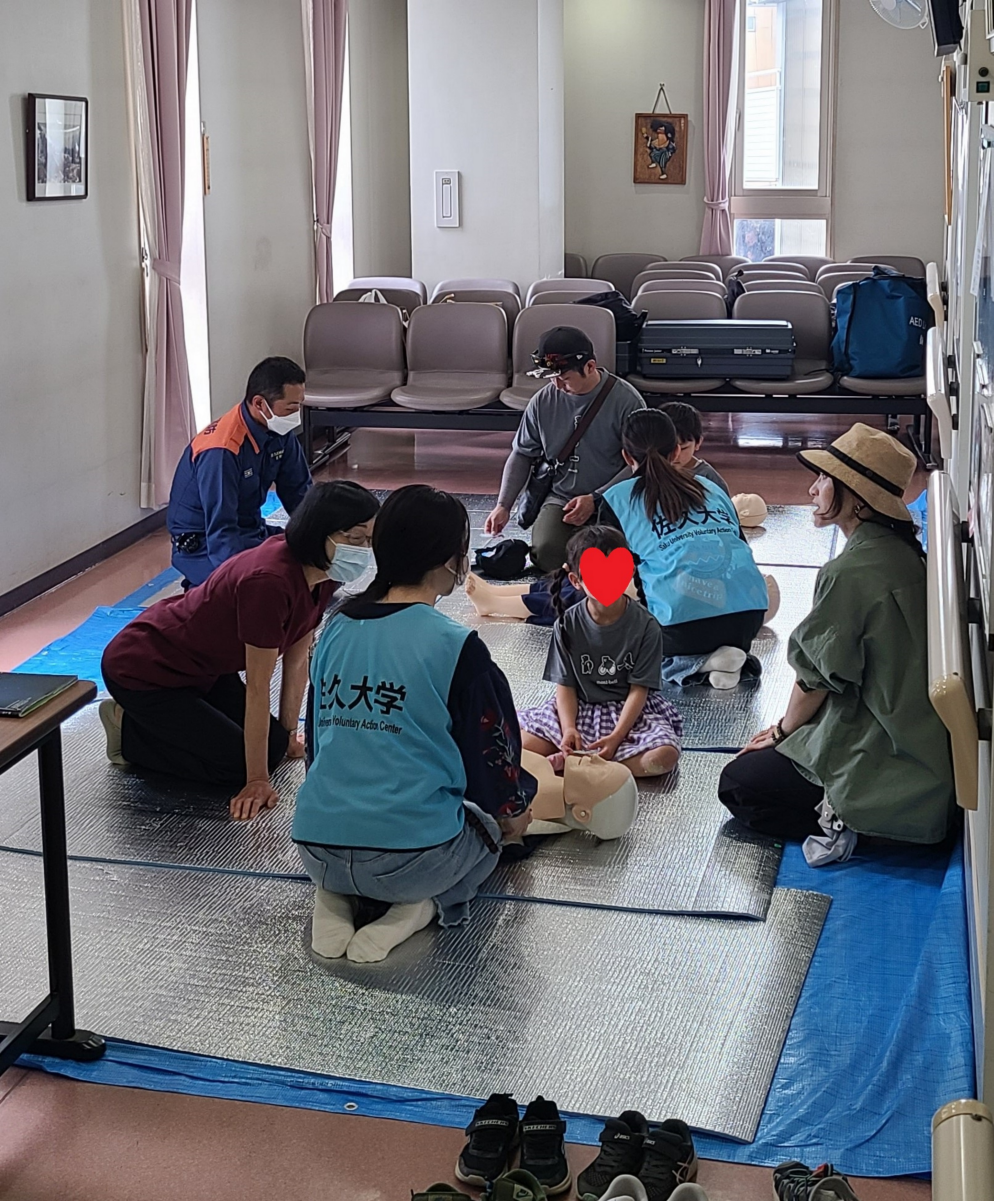
Actual CPR training scenario at the hospital festival

The study included 180 children, ranging from preschool to junior high school age, who participated in CPR training at the hospital festival on May 19, 2024. The actual training scenario is illustrated in Figure 2. As shown in Table 1, there were 91 male (51%) and 89 female (49%) respondents. Preschool children (n=59) and lower elementary school students (n=68) together accounted for 127 participants, constituting 70.6% of the total. The percentage of children who had previously learned chest compressions was 14.4%, and this percentage did not exceed 20%, even as the grade level increased.

**Table 1:** Sex and prior experience of children who received chest compression training according to school age

Groups by school age	Number (n)	Sex		Experience in Learning Chest Compression
		Male	Female	
Overall	180	51% (91/180)	49% (89/180)	14% (26/180)
Preschool children	59	56% (33/59)	44% (26/59)	10% (6/59)
Lower elementary school students	68	57% (39/68)	43% (29/68)	18% (12/68)
Upper elementary school students	46	33% (15/46)	67% (31/46)	17% (8/46)
Junior high school students	7	57% (4/7)	43% (3/7)	0% (0/7)

Table 2 shows the changes in confidence regarding chest compressions before and after the training. An improvement in confidence in performing chest compressions was observed after training, and this improvement was consistent across all age groups.

**Table 2:** Changes in confidence before and after chest compression training ^a^ The p-values were calculated using either the chi-square test or Fisher's exact test.

Groups by School Age		Very	Somewhat	Not Very	Not at all	p-value^a^
Overall	before	37	71	39	23	＜0.001
	after	95	49	9	11	
Preschool children	before	18	8	12	11	0.023
	after	28	7	3	5	
Lower elementary school students	before	17	36	11	4	＜0.001
	after	39	19	4	6	
Upper elementary school students	before	2	23	15	6	＜0.001
	after	25	19	2	0	
Junior high school students	before	0	4	1	2	0.127
	after	3	4	0	0	

Table 3 shows the changes in correct answer rates for chest compression knowledge before and after training. Knowledge of the position and depth of chest compressions was dichotomized into correct and incorrect responses. The results indicated a significant improvement in the correct answer rates across all age groups, except for the junior high school students, who had a smaller sample size.

**Table 3:** Changes in correct answer rates for chest compression knowledge before and after training ^a ^The p-values were calculated using either the chi-square test or Fisher's exact test.

Groups by School Age	Before Training		After Training	p-value^a^
	Location of chest compression,%（correct/total）			
Overall	50.6% (88/174)		97% (159/164)	<0.001
Preschool children	50.9% (27/53)		88.9% (40/45)	<0.001
Lower elementary school students	60.3% (41/68)		100% (67/67)	<0.001
Upper elementary	32.6% (15/46)		100% (46/46)	<0.001
school students				
Junior high school students	71.4% (5/7)		100% (6/6)	0.155
	Depth of chest compression,%（correct/total）			
Overall	59.2% (100/169)		92.1% (152/165)	＜0.001
Preschool children	44.9% (22/49)		70.5% (31/44)	0.013
Lower elementary school students	58.2% (39/67)		100% (68/68)	<0.001
Upper elementary	73.9% (34/46)		100% (46/46)	<0.001
school students				
Junior high school students	71.4% (5/7)		100% (7/7)	0.127

## Discussion

In this study, we evaluated the effectiveness of CPR training for children, including those of low school age, in events in which a large number of local residents participated. The results showed that only 14% of the children had previously received CPR training. Furthermore, this training increased the children's confidence and knowledge in performing chest compressions. There are concerns that younger children may not have sufficient strength to achieve an appropriate depth of chest compressions [7]. However, it has been suggested that elementary school children are highly motivated to learn and that children aged four and older can assess the initial link in the chain of survival [17, 18]. Our study also confirmed improvements in confidence and knowledge recall among children, including preschoolers, before and after the training, which supports these findings.

Receiving first aid education in early childhood is expected to increase the likelihood of retaining first aid knowledge and skills, enabling effective response in actual emergencies [19, 20]. Promoting first aid education regardless of age is important; the European Resuscitation Council (ERC) recently recommended that all schoolchildren learn CPR [12]. However, our survey revealed that only 14% of the children had previously received CPR training, indicating a lack of such initiatives.

Previous studies also support the effectiveness of CPR training in schools [7, 9]. Schools provide a setting where many people can receive education at a low cost. Utilizing schools can also increase the number of people trained in first aid. It is estimated that at least 15% of the population requires CPR training to reduce deaths due to cardiac arrest. Continuous CPR training in schools is highly important because it can attract a large number of participants.

In contrast, we conducted CPR training at a hospital festival, which is a type of health festival. While studies have demonstrated the effectiveness of CPR training for adults at health festivals [15], few reports have targeted children, including preschoolers. Based on the results of this study, a strategy for providing CPR training, including chest compressions, to children of all ages at health education festivals in addition to schools may be effective.

Generalizability of the study

The children who participated in this study were accompanied by their parents who participated in the hospital festival, a health awareness event, suggesting that they might have come from health-conscious families. However, since the hospital festival is free and attended by many local residents, with over 20,000 visitors, there is likely minimal bias in the household environment. Additionally, the survey was conducted using a sticker-based response method instead of a questionnaire, making it accessible to preschoolers and facilitating participation. Thus, the generalizability of the results is high, which is a strength of the present study. However, regarding junior high school students, the small sample size and potential background bias make it difficult to generalize the results to the entire population of junior high school students.

Limitations

This study has several limitations. First, we conducted CPR training that focused specifically on chest compressions to make it easier for preschoolers to participate, excluding Automated External Defibrillator instructions and the process of artificial respiration. Therefore, it may be difficult to compare our results with those of other studies that have used these techniques.

Second, there were only a few junior high school participants, resulting in an insufficient evaluation of this age group. Additionally, the training duration was short, 10-15 minutes. It has been suggested that to improve CPR skills in schoolchildren, 50-70 minutes of training per session and at least two hours of training annually are required [21], indicating that the training provided might have been insufficient to acquire CPR skills. However, studies have shown that even 10-minute short training sessions on dispatch-assisted CPR (DACPR) enable laypersons to start chest compressions faster in emergencies, suggesting that our training duration may not have been excessively short. Moreover, because of its short duration, training could be more efficiently provided to many participants, making it suitable for events such as hospital festivals.

Third, in this study, we chose a sticker-based response method to enhance convenience and feasibility for young children at the festival venue. However, there are several limitations to collecting information via stickers. Since it is impossible to link respondents with their responses, comparing the attributes of respondents or their answers is impossible. As a result, inappropriate responses cannot be excluded. The basis for including 180 participants was that 180 respondents answered the first question on their experience with chest compression training. However, with the method used in this study, it was impossible to exclude those who did not respond after the training. This limitation of the study might have impacted the results. On the other hand, the differences in the number of respondents before and after the training were minimal, with 170 to 164 for changes in confidence, 174 to 164 for questions on chest compression location, and 169 to 165 for questions on chest compression depth. These small differences suggest that the overall impact on the results is minimal.

Also, using a sticker-based survey method could introduce social bias, where others' responses may directly or indirectly influence an individual's answers, potentially reducing the validity of the survey results. To mitigate this effect, we implemented a system where the response area was covered with an opaque shield, preventing participants from seeing others' choices until they made their selection. As a result, we believe that this influence was significantly reduced. In addition, since individuals cannot be tracked, the study design is an ecological study targeting a group. Therefore, there is the potential for ecological fallacy, where group-level data may not apply to individuals. Future research should focus on collecting data at the individual level, even for younger children, and compare the results with those of the current study.

Moreover, this study compares responses before and after the training (intervention) but does not include a control group, making it impossible to evaluate the effects in the absence of the intervention or comparison to other interventions. This is also a limitation of the study design.

Although this study was focused on children, bias could occur because children might be influenced by adult involvement and socialized to respond in a particular way. In addition, measuring children's attitudes and beliefs is difficult and is included in the limitation of this study.

Finally, repetition is important in CPR training, with shorter intervals between training sessions enhancing CPR skills [22]. In this study, the training was conducted only once, and it is important to investigate the effects of repeated training. Additionally, as effectiveness was assessed immediately after training, long-term learning effects, i.e., retention of memory, were not examined. However, children under 12 years old have a higher capacity to recall newly acquired motor skills in the long term than adults, which may make the recall of physical skills such as CPR more effective [23].

Future cohort studies are needed to evaluate the long-term impact of training (such as skill retention and proficiency six and twelve months later) and determine whether multiple training sessions are necessary to maintain skills. Moreover, larger-scale surveys that include a wider range of children, such as junior high and high school students, are required.

## Conclusions

We conducted CPR training for children, including preschoolers, at a hospital festival and confirmed improvements in knowledge and confidence across all age groups before and after the event. Implementation of CPR training at a young age is important, and future large-scale cohort studies are needed to evaluate the long-term retention of knowledge and skills.

## References

[1] Taniguchi D, Baernstein A, Nichol G (2012). Cardiac arrest: a public health perspective. Emerg Med Clin North Am.

[2] Ho AF, Lim MJ, Earnest A (2023). Long term survival and disease burden from out-of-hospital cardiac arrest in Singapore: a population-based cohort study. Lancet Reg Health West Pac.

[3] Yan S, Gan Y, Jiang N (2020). The global survival rate among adult out-of-hospital cardiac arrest patients who received cardiopulmonary resuscitation: a systematic review and meta-analysis. Crit Care.

[4] Xu F, Zhang Y, Chen Y (2017). Cardiopulmonary resuscitation training in china: current situation and future development. JAMA Cardiol.

[5] Van Hoeyweghen RJ, Bossaert LL, Mullie A (1993). Quality and efficiency of bystander CPR. Resuscitation.

[6] Allan KS, Mammarella B, Visanji M (2023). Methods to teach schoolchildren how to perform and retain cardiopulmonary resuscitation (CPR) skills: a systematic review and meta-analysis. Resusc Plus.

[7] Tse E, Plakitsi K, Voulgaris S, Alexiou GA (2023). The role of a first aid training program for young children: a systematic review. Children (Basel).

[8] Ko JS, Kim SR, Cho BJ (2023). The effect of cardiopulmonary resuscitation (CPR) education on the CPR knowledge, attitudes, self-efficacy, and confidence in performing CPR among elementary school ctudents in Korea. Healthcare (Basel).

[9] Zenani NE, Bello B, Molekodi M, Useh U (2022). Effectiveness of school-based CPR training among adolescents to enhance knowledge and skills in CPR: a systematic review. Curationis.

[10] Tanaka H, Nakao A, Mizumoto H, Kinoshi T, Nakayama Y, Takahashi H, Shimazaki S (2011). CPR education in Japan--past, present and future (Article in Japanese). Nihon Rinsho.

[11] Hori S, Suzuki M, Yamazaki M, Aikawa N, Yamazaki H (2016). Cardiopulmonary resuscitation training in schools: a comparison of trainee satisfaction among different age groups. Keio J Med.

[12] Böttiger BW, Lockey A, Georgiou M (2020). Kids Save Lives: ERC position statement on schoolteachers' education and qualification in resuscitation. Resuscitation.

[13] Escoffery C, Rodgers KC, Kegler MC, Ayala M, Pinsker E, Haardörfer R (2014). A grey literature review of special events for promoting cancer screenings. BMC Cancer.

[14] Escoffery C, Rodgers KC, Kegler MC (2014). A systematic review of special events to promote breast, cervical and colorectal cancer screening in the United States. BMC Public Health.

[15] Nas J, Thannhauser J, Konijnenberg LS, van Geuns RM, van Royen N, Bonnes JL, Brouwer MA (2022). Long-term effect of face-to-face vs virtual reality cardiopulmonary resuscitation (CPR) training on willingness to perform CPR, retention of knowledge, and dissemination of CPR awareness: a secondary analysis of a randomized clinical trial. JAMA Netw Open.

[16] Owen A, McGeorge E (2016). A novel way to promote mass public engagement in CPR education. Resuscitation.

[17] Bollig G, Myklebust AG, Østringen K (2011). Effects of first aid training in the kindergarten--a pilot study. Scand J Trauma Resusc Emerg Med.

[18] Ammirati C, Gagnayre R, Amsallem C, Némitz B, Gignon M (2014). Are schoolteachers able to teach first aid to children younger than 6 years? A comparative study. BMJ Open.

[19] Weidenauer D, Hamp T, Schriefl C (2018). The impact of cardiopulmonary resuscitation (CPR) manikin chest stiffness on motivation and CPR performance measures in children undergoing CPR training-a prospective, randomized, single-blind, controlled trial. PLoS One.

[20] Schroeder DC, Semeraro F, Greif R (2023). Kids save lives: Basic life support education for schoolchildren: a narrative review and scientific statement from the International Liaison Committee on Resuscitation. Circulation.

[21] Kuvaki B, Özbilgin Ş (2018). School children save lives. Turk J Anaesthesiol Reanim.

[22] Oermann MH, Krusmark MA, Kardong-Edgren S, Jastrzembski TS, Gluck KA (2020). Training interval in cardiopulmonary resuscitation. PLoS One.

[23] Musselman KE, Roemmich RT, Garrett B, Bastian AJ (2016). Motor learning in childhood reveals distinct mechanisms for memory retention and re-learning. Learn Mem.

